# Molybdenum Trioxide: Efficient Nanosorbent for Removal of Methylene Blue Dye from Aqueous Solutions

**DOI:** 10.3390/molecules23092295

**Published:** 2018-09-08

**Authors:** Souad Rakass, Hicham Oudghiri Hassani, Mostafa Abboudi, Fethi Kooli, Ahmed Mohmoud, Ateyatallah Aljuhani, Fahd Al Wadaani

**Affiliations:** 1 Chemistry Department, College of Science, Taibah University, Al-Madinah 30002, Saudi Arabia; oudghiri_hassani_hicham@yahoo.com (H.O.H.); abboudi14@hotmail.com (M.A.); caadil77@yahoo.co.uk (A.M.); ateyatallah@hotmail.com (A.A.); fwadaani@taibahu.edu.sa (F.A.W.); 2 Département de Chimie, Faculté des Sciences Dhar El Mahraz, Université Sidi Mohamed Ben Abdellah, B. P. 1796 (Atlas), Fès 30003, Morocco; 3 Community College, Taibah University-Al-Mahd Branch, Al-Mahd 42112, Saudi Arabia; fethi_kooli@yahoo.com

**Keywords:** α-MoO_3_, nanosorbent, methylene blue, removal, regeneration

## Abstract

Nano Molybdenum trioxide (α-MoO_3_) was synthesized in an easy and efficient approach. The removal of methylene blue (MB) in aqueous solutions was studied using this material. The effects of various experimental parameters, for example contact time, pH, temperature and initial MB concentration on removal capacity were explored. The removal of MB was significantly affected by pH and temperature and higher values resulted in increase of removal capacity of MB. The removal efficiency of Methylene blue was 100% at pH = 11 for initial dye concentrations lower than 150 ppm, with a maximum removal capacity of 152 mg/g of MB as gathered from Langmuir model. By comparing the kinetic models (pseudo first-order, pseudo second-order and intraparticle diffusion model) at various conditions, it has been found that the pseudo second-order kinetic model correlates with the experimental data well. The thermodynamic study indicated that the removal was endothermic, spontaneous and favorable. The thermal regeneration studies indicated that the removal efficiency (99%) was maintained after four cycles of use. Fourier Transform Infrared (FTIR) and Scanning Electron Microscopy (SEM) confirmed the presence of the MB dye on the α-MoO_3_ nanoparticles after adsorption and regeneration. The α-MoO_3_ nanosorbent showed excellent removal efficiency before and after regeneration, suggesting that it can be used as a promising adsorbent for removing Methylene blue dye from wastewater.

## 1. Introduction

Dyes are organic pollutants that have a complex chemical structure, are highly stable; resist washing, light and microbial invasions and poorly biodegradable [1,2,3,4]. They are harmful to aquatic life and humans and their removal is of significant importance [5,6,7,8].

Several methods were performed for dye removal from industrial effluents and wastewater including flocculation, coagulation, adsorption, ion exchange, membrane separation, photodegradation, extraction, chemical oxidation and biological treatment [9,10,11,12,13,14,15]. Adsorption proposes the advantages of effectiveness, simplicity and low cost from among those above-mentioned approaches. [1,16,17,18,19,20,21].

Several natural and synthetic substances were reported earlier in the literature as adsorbents for organic dyes [22,23,24,25,26,27,28,29,30,31]. The adsorption performance of biosorbents is usually restricted by the low surface area, which results in low adsorption capacities [32]. Activated carbon (AC), from agricultural and solid wastes as the nontoxic and easily available adsorbent, is considered as a general adsorbent for removing pollutants such as organic dyes from wastewater due to its porous structure, high surface areas, fast adsorption kinetics, large adsorption capacities and general material as a support for loading nanomaterials [33,34]. However, AC is still considered highly expensive based on the market price of the commercial activated carbon available. In addition, its poor mechanical and regeneration properties have limited its use in the adsorption process. [21,28,29].

Recently, nanomaterials as synthetic adsorbents have attracted a lot of research interest because of their distinctive properties such as electron conduction, large surface area, highly active sites, low mass used and the ability to modify their surface properties [35,36]. The nanomaterials are grouped in different categories such as metal oxide, carbonaceous, bio or magnetic nanomaterials. They have been widely studied as removal agents for dyes [3,5,22,27,30,35,36,37,38,39,40]. Some examples of metal oxides nanomaterials used for dyes removal are Titanium dioxide [41], Zinc oxide [42], Magnesium oxide [43] and Magnetic iron oxide [14].

The nanoparticles are synthesized by various methods, which are categorized as three types, namely chemical, physical and mechanical processes [44]. The chemical process involves the use of chemistry solutions, making this process, not suitable for large scale production, due to its high expenses and slow to manufacture [45,46,47].

Molybdenum can be found in several oxide stoichiometries, which have been used for a variety of high-value research and commercial applications [48]. Furthermore, MoO_3_ is a polymorph material with at least four known phases monoclinic (β-MoO_3_), orthorhombic (α-MoO_3_), high pressure monoclinic (MoO_3_-II) and hexagonal (h-MoO_3_) [49,50,51,52]. Due to the outstanding electrochemical and catalytic activities, α-MoO_3_ has been widely considered [48,53,54]. Thus far, a number of α-MoO_3_ nanostructures were synthesized including nanobelts, nanoparticles, nanosheets, flower-like hierarchical structures and nanoflakes [49,55,56,57,58,59,60,61,62,63]. However, few studies are reported on the use of Molybdenum trioxide for removing dyes. Beltran et al. [64] reported that hexagonal and orthorhombic phases of MoO_3_ nanoparticles synthesized using microwave radiation followed by high-energy mechanical milling were used for Methylene blue (MB) removal. Approximately a 98% of MB was removed from 20 ppm content in water, without using photon radiation in about 25 min [64]. Huge challenge is seeking to the development of nanomaterials, easily synthetized and presenting high performance criteria for removal of dyes and regeneration [22,36,65].

In our previous work, Molybdenum trioxide (α-MoO_3_) nanorods and stacked nanoplates were synthesized easily and efficiently at a rather low temperature with the use of a simple and economical approach [61,66]. In this study, the capacity of the materials of interest were tested to remove methylene blue dye (MB) from aqueous solutions. The methylene blue dye is classified as a prior pollutant due to its broad usage in various industrial applications, for example coloring agents for cotton, leather, wool and silk and so forth [67]. For this purpose, the effect of a variety of parameters such as adsorbent dose, contact time, pH, initial dye concentrations and temperature were evaluated. The thermodynamic and kinetic studies were performed. The experimental equilibrium data was examined using Temkin, Freundlich, Langmuir and Dubinin–Radushkevich models. Thermal regeneration of α-MoO_3_ nanosorbent was also studied.

## 2. Experimental

### 2.1. Preparation of Molybdenum Trioxide Nanosorbent

All chemicals were purchased from Sigma-Aldrich (St. Louis, MO, USA) and used as received without any changes, except for the methylene blue (MB) dye, which was supplied by Panreac, Barcelona, Spain.

Molybdenum trioxide nanosorbent (α-MoO_3_) was synthesized using the thermal decomposition of an oxalic precursor of Molybdenum gained from the reaction of oxalic acid and ammonium molybdate (NH_4_)_6_Mo_7_O_24_·4H_2_O in the solid state, as described in our earlier work [61]. Oxalic acid and ammonium molybdate (NH_4_)_6_Mo_7_O_24_·4H_2_O were mixed together in a ratio of Mo:acid of 1:3. The mixture was ground then heated on a hot plate at 160 °C. Then, the oxalic precursor was decomposed at 350 °C in a tubular furnace open on both ends.

### 2.2. Adsorption Experiments

The removal of MB was carried out by batch adsorption experiments [68]. The removal of MB by α-MoO_3_ was carried out by stirring specific amount of adsorbent into 100 mL of MB solution of known concentrations at specific temperature (T = 25, 50 and 70 °C) and at different contact times (10, 30, 60, 90 and 120 min). At the end of predetermined time intervals, the solution was filtrated with a 0.45 µm syringe filter (Whatman, Sigma-Aldrich, St. Louis, MO, USA) and examined using a UV-Visible spectrometer (Thermo Fisher Scientific, Madison, WI, USA) at λ_max_ = 665 nm. The pH of the MB solution was adjusted by adding either 0.01 N NaOH or 0.01 N HCl solutions. The percentage of removal (%) and the removed amount of MB at equilibrium *q_e_* (mg/g) were calculated using the following relationships.

(1) $$\mathrm{Removal}\;\%=\frac{C_{i}-C_{f}}{C_{i}}\times 100$$

(2) $$q_{e}=\frac{\left(C_{i}-C_{f}\right)}{M}\times V$$

where C_i_ and C_f_ represent the initial and equilibrium concentration of MB (ppm), respectively. V is the used volume of solution (L) and M is the added mass of α-MoO_3_ (g). The results were repeated three times and the uncertainty was about 3%.

### 2.3. Adsorbent Regeneration Method

For the regeneration experiments, a solution of 150 ppm was used and the removal equilibrium time was extended for 2 h. The fresh spent α-MoO_3_ was filtered, dried at 100 °C and calcined at 400 °C for 1 h, under air atmosphere. The calcined α-MoO_3_ was tested again at the same conditions. The regeneration process was repeated for three cycles. 

### 2.4. Characterization

The powder characterization in terms of the phase composition of the synthetized α-MoO_3_ nanosorbent, was analyzed by XRD (X-ray diffractometer 6000, Shimadzu, Tokyo, Japan, installed with λ_Cu-Kα_ = 1.5406 Ǻ and Ni filter). The specific surface area was deduced from the nitrogen isotherm adsorption and using the BET equation (D_BET_ = 6000/d.S, where S is the specific surface area and d is the density), as reported in our previous work [61]. The specific surface area value was 41.02 m^2^/g.

The presence of MB dye on the α-MoO_3_ nanoparticles after the adsorption and regeneration studies was confirmed by FTIR spectroscopy using IR Affinity-1S Shimadzu apparatus (Shimadzu, Tokyo, Japan) in the range of 400 and 4000 cm^−1^ using KBr pellets. Scanning electron microscope (SEM) analysis was performed using Quanta Feg 250 (Thermo Fisher Scientific, Hillsboro, OR, USA). The concentration at equilibrium was determined using UV-Visible spectrophotometer (Thermo Scientific Genesys 10S, Madison, WI, USA).

## 3. Results and Discussion

### 3.1. Removal of MB

#### 3.1.1. Effect of Initial Dye Concentration and Contact Time

**Figure 1:**
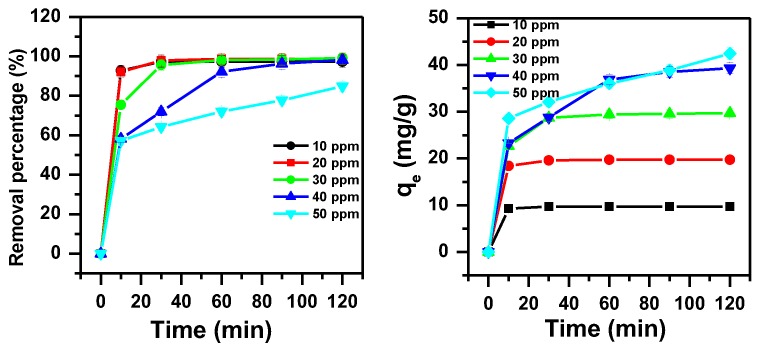
Effect of initial dye concentration and contact time on removal efficiency of methylene blue (MB) using α-MoO_3_ (m_adsorbent_ = 0.1 g, T = 25 °C, pH = 5.5).

The effect of contact time and initial dye concentration on the removal of MB dye was studied and presented in Figure 1. The removal of MB increases with the increase of contact time and reaches a maximum value of 99% at about 30 min for initial MB concentrations of 10, 20 and 30 ppm and 120 min of contact time for initial dye concentration of 40 ppm. The removal capacity was improved from 19 mg/g to 42 mg/g when the initial dye concentrations increased from 20 ppm to 50 ppm, respectively. These results can be clarified by the primarily great availability of vacant sites on the α-MoO_3_ surface, which steadily decreases as the sites are filled up over time as a result of the sorption process [69].

#### 3.1.2. Effect of Adsorbent Dose and Initial Dye Concentration

**Figure 2:**
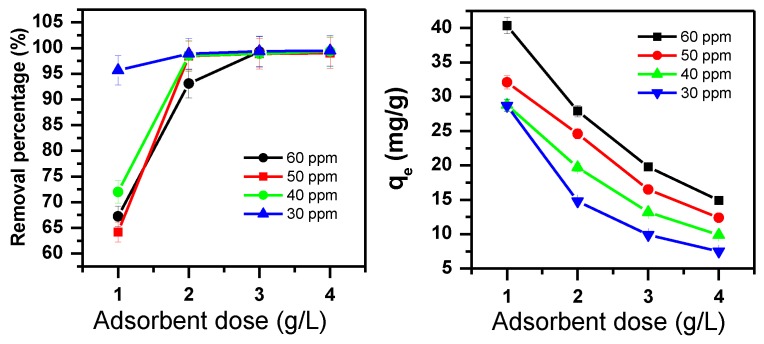
Adsorbent dose effect and initial dye concentration for the efficiency of MB removal using α-MoO_3_ for 30 min, T = 25 °C, pH = 5.5.

The adsorbent dose is a very important parameter in the adsorption process [70]. The removal of MB using α-MoO_3_ was investigated by varying the adsorbent dose from 1.0 to 4.0 g/L and the initial dye concentrations from 30 to 60 ppm (Figure 2).

For lower initial concentrations less than 50 ppm, 2 g/L of adsorbent dose was needed to achieve 99% of MB removal percentage. However, for 60 ppm, 3 g/L was the minimum adsorbent needed to obtain 99% of removal efficiency.

The amount of MB removed decreased with respect to an increase of adsorbent dose and this is shown in Figure 2. This is due to the increase of the available active sites on the adsorbents’ surface area. These results can be explained by the availability of more active sites as the adsorbent dose increased [70].

#### 3.1.3. Temperature Effect

**Figure 3:**
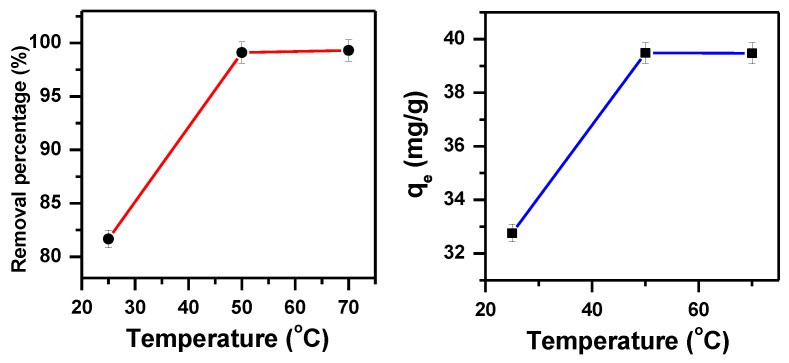
Effect of temperature on the removal efficiency of 40 ppm of MB solution using α-MoO_3_ (t = 30 min, pH = 5.5).

As the temperature has a great effect on removing dyes [71], an investigation was carried out on temperature as a parameter on its own from 25 to 70 °C during the process of removing the MB dye, this can be seen in Figure 3. The percentage removal of MB (at C_i_ = 40 ppm) has gone up from 82% to 99% and the removal capacity has increased from 33 mg/g to 39 mg/g. In actual fact, the removal activity of the adsorbent sites enhanced as the temperature increased giving rise to the dye molecule motion [71,72].

**Figure 4:**
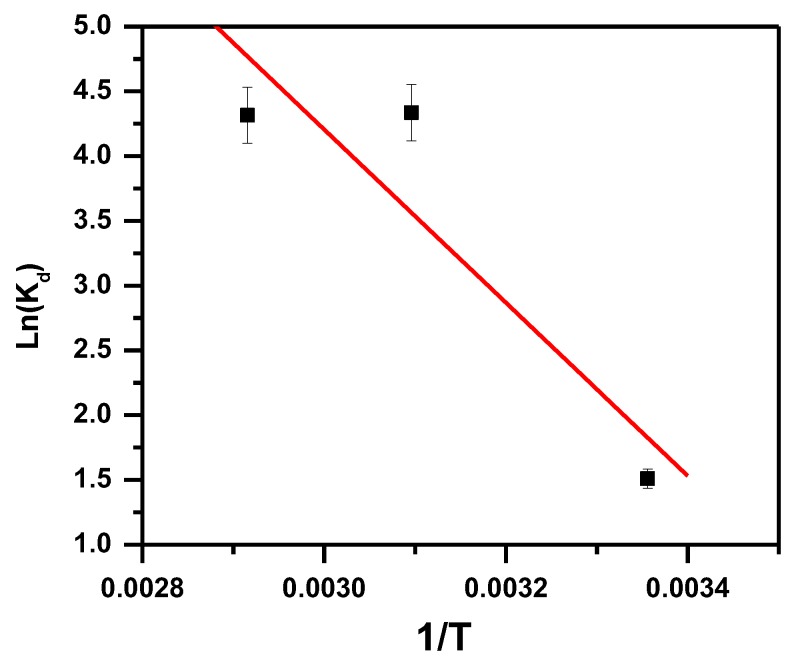
Von’t Hoff plot showing the temperature effect for the removal of MB by α-MoO_3._

Thermodynamic factors are important in the adsorption process [73,74]. The likelihood and the mechanism of adsorption can be projected in reference to the thermodynamic factors [73]. Thermodynamic parameters can be deduced using the following equations:

(3) $$\Delta G^{o}=-{\mathrm{RTLnK}}_{d}$$

(4) $$K_{d}=\frac{C_{a}}{C_{e}}$$

(5) $${\mathrm{LnK}}_{d}=\frac{\Delta S^{o}}{R}-\frac{\Delta H^{o}}{\mathrm{RT}}$$

where R is the gas constant (J·mol^−1^·K^−1^), ΔG° is the free energy (KJ·mol^−1^), K_d_ is the distribution constant, T is absolute temperature (K), C_e_ is the equilibrium concentration (mol/L), C_a_ is the amount of dye adsorbed on the adsorbent at equilibrium (mol/L), ΔH° is the standard enthalpy (KJ·mol^−1^) and ΔS° is the standard entropy (KJ·mol^−1^·K). ∆S° and ∆H° values were achieved from the intercept and slope of plot lnK_d_ versus 1/T and presented in Figure 4 (The value of the regression correlation coefficients (R^2^) is 0.83). ∆G° values were obtained from Equation (3) and presented in Table 1. The adsorption is favorable and spontaneous, indicated by the negative value of ∆G°. ∆H° value indicates that MB removal occurred in a physisorption process as indicated by the positive value of ∆H° (90 KJ mol^−1^) [75]. The increased disorder and randomness at the solid solution interface of MB and α-MoO_3_ is indicated by the positive values of ∆S°. The adsorbed water molecules are displaced by the adsorbate molecules and therefore more translational energy is gained than is lost, this leads the system occurring randomly [76].

#### 3.1.4. Effect of pH

**Figure 5:**
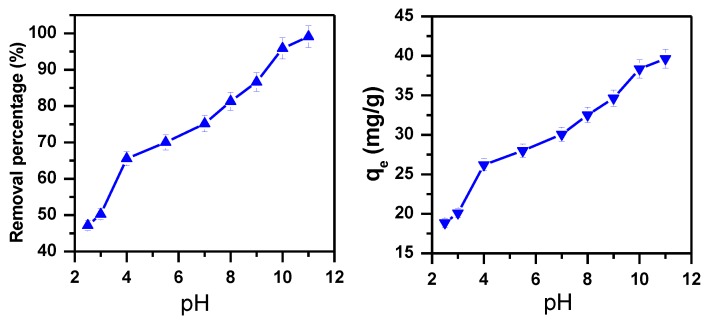
Effect of pH on the removal efficiency of 40 ppm of MB solution using α-MoO_3_ (m_ads_ = 0.1 g, T = 25 °C, t = 30 min).

pH is an essential element that controls the removal of dyes [71]. Consequently, the effect of pH for the removal of MB using α-MoO_3_ nanosorbent was studied by variable pH values from 2.5 to 11 at temperature of 25 °C and initial concentration of 40 ppm. As presented in Figure 5, the MB removal is evidently pH dependent. The percentage removal increases from 47% to 99% as pH increases from 2.5 to 11. The amount of dye removed per unit mass of adsorbent at equilibrium (q_e_) increased from 19 to 40 mg/g by variation of pH from 2.5 to 11. At pH = 11 the hydroxyl group (OH^−^) in solution favors the positive charge of the MB since its pKa equals 3.8 [77]. Therefore, pH = 11 was considered as the optimum value for MB removal using α-MoO_3_ nanosorbent.

#### 3.1.5. Effect of MB Initial Dye Concentration and Contact Time after pH Adjustment

**Figure 6:**
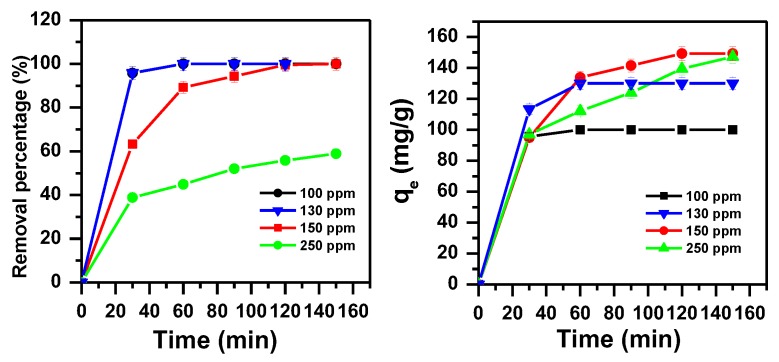
Effect of initial dye concentration contact time on the removal efficiency of MB using α-MoO_3_ at pH 11 (m_ads_ = 0.1 g, T = 25 °C).

The removal efficiency of α-MoO_3_ was examined for higher concentrations of methylene blue dye at pH = 11 as presented in Figure 6. Interestingly, the percent of removal of MB was 100% after 60 min and 120 min for initial dye concentrations of 100 and 150 ppm, respectively. The removed amount of MB was 100 mg/g for initial dye concentrations of 100 ppm and 150 mg/g for initial dye concentrations of 150 and 250 ppm.

### 3.2. Kinetic Study

The kinetic models based on the removal capacity were fitted to experimental data to determine the rates of adsorption for MB dye molecules and to investigate the mechanism of the removal process [78].

The data obtained from the kinetics of removing MB using 0.1 g of α-MoO_3_ nanosorbent at room temperature and pH = 11 was analyzed by pseudo first-order (PFO), pseudo second-order (PSO) and intraparticle diffusion (IPD) kinetic models. The equations of the studied models are given in Table 2.

**Figure 7:**
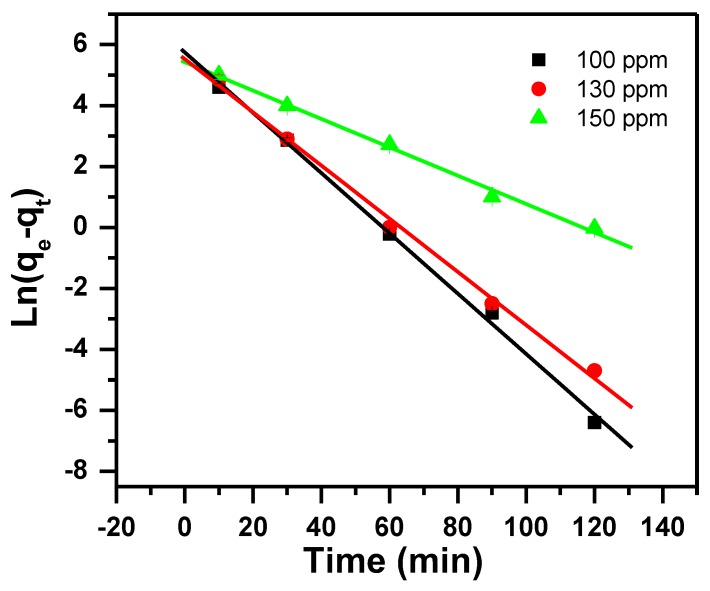
Pseudo first-order model plot showing the effect of contact time and initial dye concentration of MB removal by α-MoO_3_.

**Figure 8:**
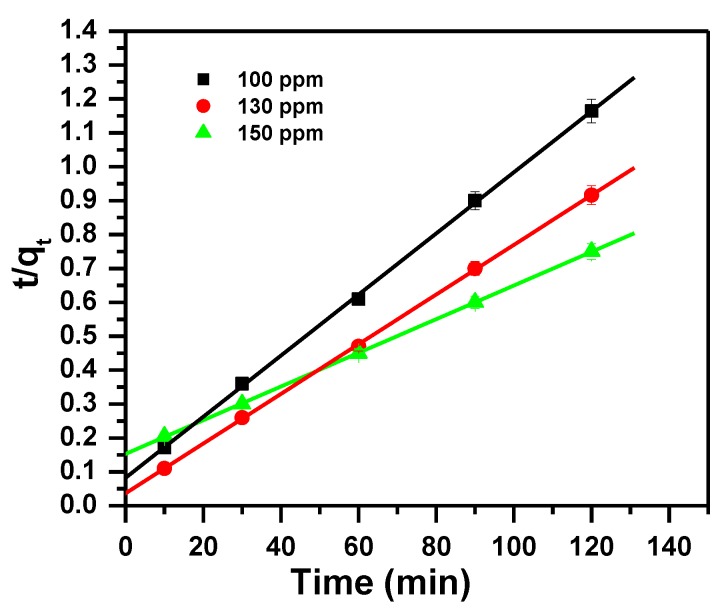
Pseudo Second order model plot showing the effect of contact time and initial dye concentration of MB removal by α-MoO_3_.

**Figure 9:**
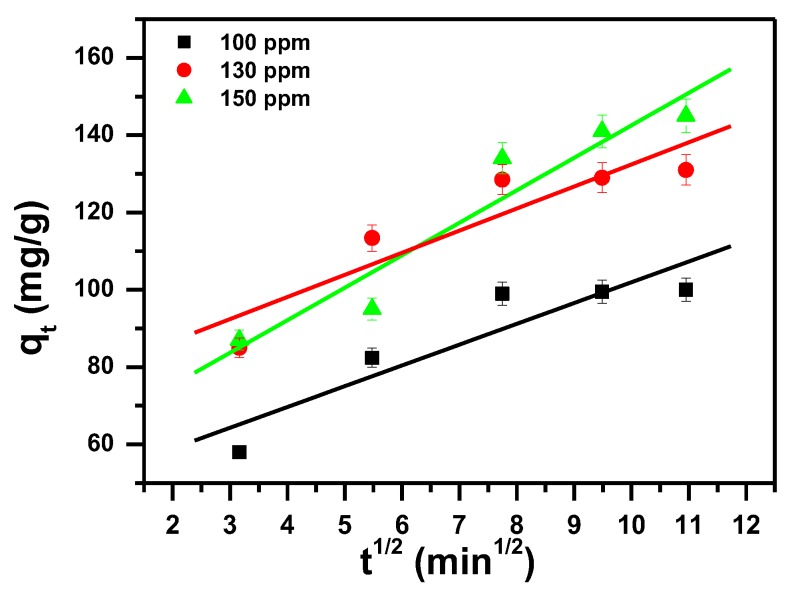
Intra-particle diffusion model plot showing the effect of contact time and initial dye concentration of MB removal by α-MoO_3_.

The three model parameters, pseudo first, pseudo second and intra-particle diffusion are tabulated in Table 3 and presented in Figure 7, Figure 8 and Figure 9 respectively. The three models differ in their regression correlation coefficients (R^2^). Pseudo first ranges from 0.995 to 0.997, whereas Pseudo second is 0.998 to 1.000 and intra-particle is 0.832 to 0.910, with their different concentrations used. The R^2^ for pseudo second-order is close to 1 and hence this model fitted well the experimental data.

### 3.3. Adsorption Isotherms

To optimize the design of a removal system for the MB molecules, various isotherm equations have been used to describe the equilibrium characteristics of the removal process [81]. Four adsorption models were investigated, namely Freundlich, Langmuir, Temkin isotherm and Dubinin–Radushkevich models. The equations for the four tested models are summarized in Table 4.

**Figure 10:**
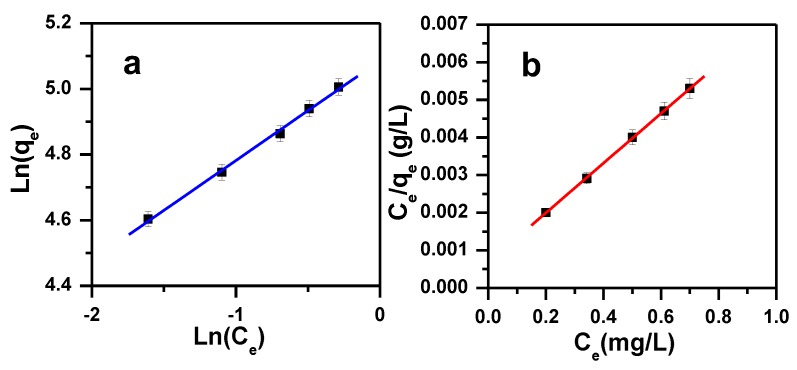
Freundlich (**a**) and Langmuir (**b**) isotherm model plots showing the effect of initial dye concentration for the removal of MB by α-MoO_3._

Langmuir, Freundlich, D–R isotherm and Temkin models were applied to fit the experimental data. The values of the regression correlation coefficients (R^2^) and the model parameters are included within Table 5 and shown in Figure 10. Langmuir equation showed the highest value of R^2^ (1.000) and D–R model showed the lowest value of R^2^ (0.939), whereas intermediate values were achieved for Temkin and Freundlich (0.989 and 0.997 respectively). Langmuir model fits wells with the experimental data and the MB removal took place on homogenous surface forming a monolayer on the α-MoO_3_ adsorbent, with high adsorption capacity of 152 mg/g. MB dye removal by α-MoO_3_ is favorable which is indicated by the separation factor R_L_ ranging from 0.0007 to 0.0090.

The comparative links between α-MoO_3_ and other sorbents presented in this work are shown in Table 6. The Molybdenum trioxide (α-MoO_3_) nanorods and stacked nanoplates synthesized easily and efficiently at rather low temperature with the use of simple and economical approach [61,66] showed high removal capacity. In addition, the molybdenum trioxide is presenting the advantage to be successfully regenerated as it will be presented in this paper. Moreover, no modification is needed for the molybdenum trioxide because it is used as prepared which is not the case when using supported gold nanoparticles or when using nanotubes. Another important point to raise is that the mass production of the MoO_3_ is possible as the production can be done easily at higher scale.

### 3.4. Regeneration and Characterization of the Nanosorbent

#### 3.4.1. Regeneration Efficiency

The regeneration and repeatability of the adsorbent are very critical for the practical application. Many regeneration procedures were proposed in the literature survey, including thermal treatment, chemical extraction, bio-regeneration, supercritical regeneration, microwave irradiation and so forth. Thermal regeneration is often applied for regeneration of exhausted activated carbon [91]. In our case, the structure of α-MoO_3_ removal agent was stable and the thermal treatment method was selected in this part.

**Figure 11:**
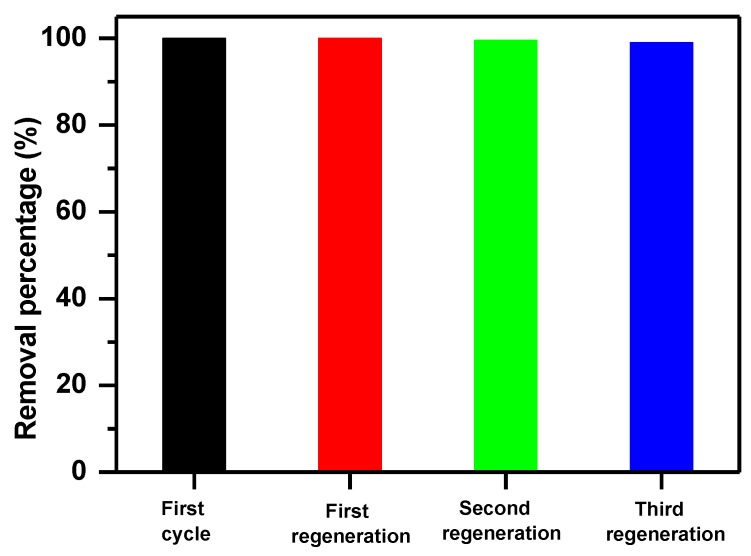
Recycled efficiency of α-MoO_3_ for removal of Methylene blue.

It is found that α-MoO_3_ could be regenerated through thermal treatment. The MB removal efficiency of α-MoO_3_ was maintained after three cycles of regeneration with an average of 99% as presented in Figure 11. The high removal efficiency indicated that the regeneration of the adsorbent by calcination under air atmosphere at 400 °C was highly efficient and suggesting an excellent reusability.

#### 3.4.2. Fourier-Transform Infrared Spectroscopy

**Figure 12:**
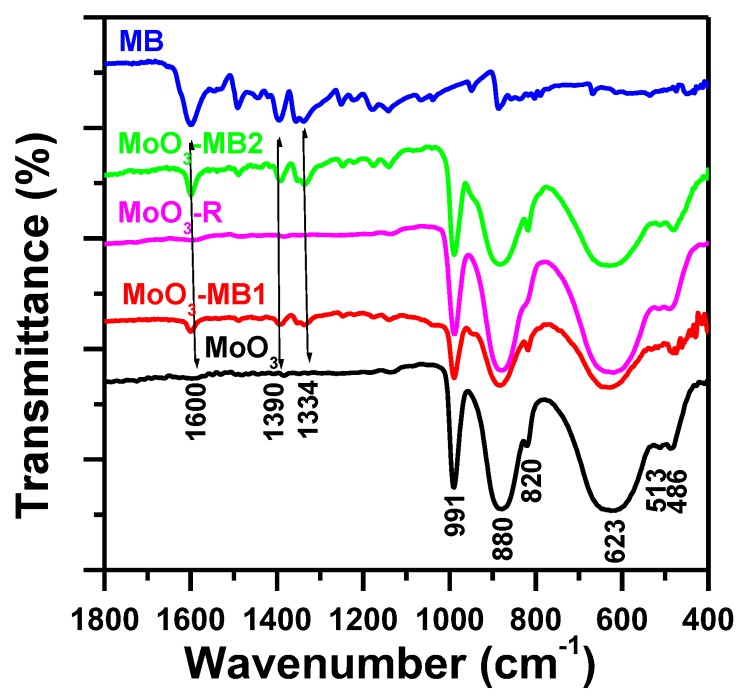
Fourier transform infrared (FTIR) spectra of MoO_3_, MoO_3_-MB1, MoO_3_-R, MoO_3_-MB2 and MB.

In order to fully recognize the MB removal process by α-MoO_3_ nanosorbent, the materials exposed to MB were studied by IR spectroscopy. Figure 12 shows the FTIR spectra of the α-MoO_3_ sample before and after removal of MB dye. As seen, the characteristic stretching and flexing vibrations of the metal–oxygen bonds at 991, 880, 820, 513, 486 and a broad centered at 623 cm^−1^**,** corresponded to Molybdenum trioxide [92]. The FTIR spectrum of pure MB exhibited bands between 1700 and 1000 cm^−1^ [93]. While, the FTIR spectrum of α-MoO_3_ after adsorption of MB (MoO_3_-MB1) exhibited additional bands located at 1600 cm^−1^, related to C=C stretching of MB, due to the presence of MB attached to the active sites of α-MoO_3_ [94]. The FTIR spectrum of the regenerated α-MoO_3_ (MoO_3_-R) after thermal treatment was similar to the fresh α-MoO_3_. The reused sample (MoO_3_-MB2) exhibited again all bands characteristic of the MB [93]. The obtained spectrum confirmed the efficiency of the reused adsorbent.

#### 3.4.3. Scanning Electron Microscope (SEM) Analysis

**Figure 13:**
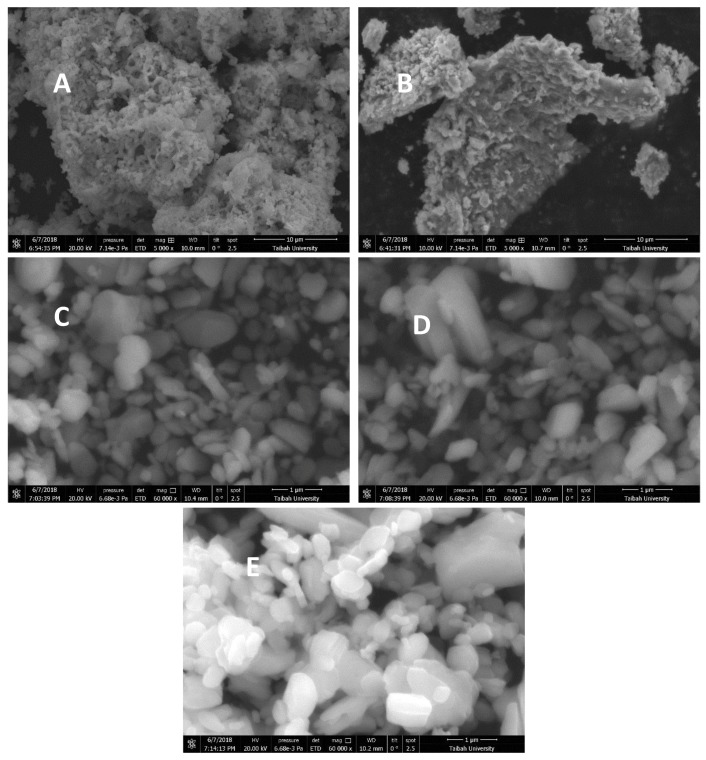
Scanning electron microscopy (SEM) Micrographs of the starting (**A**) Molybdenium trioxide (α-MoO_3_) (magnification of ×5000, scale bar of 10 μm), (**B**) after MB dye removed (magnification of ×5000, scale bar of 10 μm), (**C**) relates to the regenerated α-MoO_3_ (magnification of ×60,000, scale bar of 1 μm) and (**D**) after first regeneration/removal cycle of MB dye (magnification of ×60,000, scale bar of 1 μm), (**E**) shows the morphology of α-MoO_3_ after second regeneration process (magnification of ×60,000, scale bar of 1 μm).

It is interesting to follow up the evolution of the α-MoO_3_ morphology at different steps of the adsorption test. The SEM micrograph in Figure 13A indicated that the α-MoO_3_ particles exhibited sponge like structure, of dimensions varying from 5 to 10 microns. After removal of MB molecules, the sponge-like structure vanished and the pores were stuffed by the removed molecules (Figure 13B). Figure 13C,D indicated that the morphology of the sample was not altered after regeneration and the first reuse. In both cases the particles are less agglomerated with aggregates less than 1 micron in size. In overall, the morphology of α-MoO_3_ was not significantly modified even after the second reuse in Figure 13E.

#### 3.4.4. Removal Mechanism of MB

**Figure 14:**
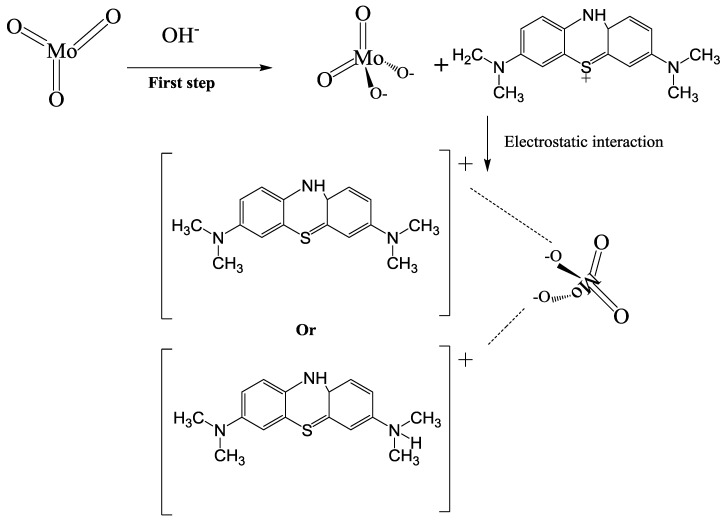
Schematic mechanism of the MB removal using the Molybdenum trioxide nanosorbent.

It was found that the removal of MB by α-MoO_3_ nanoparticles was by adsorption mechanism. In fact, the FTIR spectroscopy indicated that the removed MB cations caused by adsorption process, without chemical decomposition of MB and no intermediate compounds were detected. In addition, the increase on the effectiveness of the removal of MB using α-MoO_3_ nanoparticles by increasing the pH until 11 could be attributed to the basic media. From this establishment, a mechanism could be suggested (Figure 14). In fact, in the first step at pH = 11, the positive charge of the MB is maintained since its pKa is equal to 3.8 [77]. In addition, the hydroxyl groups (OH^−^) in the solution react with α-MoO_3_ to produce the ion molybdate (MoO_4_^2−^) without intermediate compounds [95]. Thus, the adsorption is governed by strong electrostatic interactions between the negatively surface charge of molybdate (MoO_4_^2−^) and the positively charged MB cations.

The specific surface area of α-MoO_3_ deduced from the monolayer capacity (q_m_) at natural pH and has been calculated from the following equation:

Specific Surface Area (SSA) = q_m_ × N × A (6)

where q_m_ is the monolayers capacity in moles per gram; N is Avogadro number (6.019 × 10^23^) and A is area per molecule on the surface.

The value of (57 m^2^/g) was slightly higher than the value deduced from the BET equation (42 m^2^/g), using the N_2_ adsorption isotherm. The difference between these values was related to the mechanism of adsorption related to nitrogen and MB molecules [96]. In the N_2_ absorption method, the molecules are attracted to the surface by van der Waals forces (physisorption) and multiple layers may form. However, in the case of MB used as probe molecule, there is a high bonding energy (ionic Coulombian attraction—chemisorption) and it is generally limited to a monolayer [97].

## 4. Conclusions

Nanocrystalline α-MoO_3_, synthesized through a simple method, was tested as a Nanosorbent for the removal of cationic Methylene blue dye from aqueous solution. The material exhibited higher removal efficiency (99%) at pH = 11 and a maximum removal capacity of 152 mg/g. The adsorbent was easily regenerated by calcination and the removal efficiency was 99% after three regeneration/removal cycles. Considering the easy and low-cost of α-MoO_3_ synthesis process, the high removal efficiency and its regeneration after several cycles, the synthesized α-MoO_3_ adsorbent will be proposed as promising candidate for the removal of MB from aqueous solutions.

## Figures and Tables

**Table 1:** Thermodynamic parameters for removal of MB by α-MoO_3._

Adsorbent	Adsorbate	∆H° (KJ·mol^−1^)	∆S° (KJ·mol^−1^·K)	∆G° (KJ·mol^−1^)			
α-MoO_3_	MB	90	0.316	298 K	323 K		343 K
				−3.741		−11.643	−12.305

**Table 2:** Kinetic models’ equations.

Model	Equation	Parameters
Pseudo first-order (PFD) [79]	$\mathrm{Ln}\left(q_{e}-q_{t}\right)={\mathrm{Lnq}}_{e}+K_{1}t$	q_t_: the removal capacity at time t (mg/g); q_e_: the removal capacity at equilibrium (mg/g); K_1_: the rate constant of pseudo first-order adsorption (1/min)
Pseudo second-order (PSD) [79]	$\frac{t}{q_{t}}=\frac{1}{K_{2}q_{e}^{2}}+\frac{t}{q_{e}}$	q_t_: the removal capacity at time t (mg/g); q_e_: the removal capacity at equilibrium (mg/g); K_2_: the pseudo second-order rate constant (g·mg^−1^·min^−1^)
Intraparticle diffusion (IPD) [80].	$q_{t}=K_{I}t^{0.5}+l$	I (mg/g) and K_I_ (mg/(g·min^0.5^)) are the intraparticle diffusion constants, q_t_: the removal capacity (mg/g) at time t; t: the contact time (min)

**Table 3:** Kinetic parameters for removal of MB using α-MoO_3_.

Dye C_i_ mg/L	Pseudo First-Order				Pseudo Second-Order			Intra-Particle-Diffusion Model		
	q_exp_ (mg/g)	q_e_ (mg/g)	k_1_ (1/min)	R_1_^2^	q_e_ (mg/g)	k_2_ (g/mg min)	R_2_^2^	I (mg/g)	k_i_ (mg/g min^0.5^)	R_3_^2^
100	99.8	281	0.097	0.997	111	0.00097	0.998	60.20	4.42	0.832
130	129.5	321	0.097	0.996	136	0.00147	0.999	93.15	4.03	0.834
150	149.6	225	0.045	0.995	200	0.00017	1	34.35	11.75	0.910

Where q_exp_ is the removal capacity (mg/g) at 120 min.

**Table 4:** Adsorption Isotherm model equations for removal of MB using α-MoO_3_.

Model	Equation	Parameters
Freundlich [81]	${\mathrm{Lnq}}_{e}={\mathrm{Lnq}}_{F}+\frac{1}{n}{\mathrm{LnC}}_{e}$	n: the heterogeneity factor (g/L); q_F_: the Freundlich constant (mg^(1−1/n)^·L^1/n^·g^−1^); C_e_: concentration of MB at equilibrium (ppm); q_e_: the MB dye amount adsorbed by α-MoO_3_ at equilibrium (mg/g)
Langmuir [82]	$\frac{C_{e}}{q_{e}}=\frac{1}{q_{m}K_{L}}+\frac{C_{e}}{q_{m}}$	C_e_: concentration of MB at equilibrium (ppm); q_e_: the MB dye amount adsorbed by α-MoO_3_ at equilibrium (mg/g); K_L_: Langmuir constant of adsorption (L/mg); q_m_: the maximum amount of MB dye removed by α-MoO_3_ (mg/g)
	$R_{L}=\frac{1}{1+K_{L}C_{i}}$	K_L_: the Langmuir constant; C_i_: the initial concentration of MB; R_L_: values indicate that the removal of MB could be linear (R_L_ = 1), irreversible (R_L_ = 0), favorable (0 < R_L_ < 1), or unfavorable (R_L_ > 1).
Dubinin–Radushkevich (D-R) [83]	${\mathrm{Lnq}}_{e}={\mathrm{Lnq}}_{m}-K\varepsilon^{2}$	ε: the Polanyi potential; K: constant for the sorption energy (mol^2^/kJ^2^); R: the Universal gas constant (8.314 J.mol^-1^ K^−1^); T : the temperature (K); C_e_: the equilibrium concentration of the MB dye left in the solution (ppm); q_m_: the theoretical saturation capacity.
	$\varepsilon =RTLn\left(1+\frac{1}{C_{e}}\right)$	
Temkin [84]	$q_{e}=B_{T}{\mathrm{LnA}}_{T}+B_{T}{\mathrm{LnC}}_{e}$	B_T_ = R_T_/b_T;_ b_T_: the Temkin constant related to heat of sorption (J/mol); A_T_: the Temkin isotherm constant (L/g); R: the gas constant (8.314 J/mol K); T: the absolute temperature (K)

**Table 5:** Isotherm parameters for removal of MB using α-MoO_3._

Langmuir				Freundlich			Temkin			Dubinin–Radushkevich		
q_m_ (mg/g)	K_L_ (L/mg)	R^2^	Range R_L_	q_F_ (mg^(1−1/n)^·L^1/n^·g^−1^)	1/n	R^2^	A_T_ (L/g)	B_T_ (J/mol)	R^2^	q_m_ (mg/g)	R^2^	E (KJ/mol)
152	9.58	1	0.0007–0.0090	161	0.301	0.997	74.8	36.56	0.989	152	0.939	16

**Table 6:** Earlier reports for the highest amount of MB removed (q_m_).

Nanosorbent	Q_max_ (mg/g)	Reference
Magnetic iron oxide nanosorbent	25.54	[14]
Alkali-activated multiwalled carbon nanotubes	399.00	[85]
Fe_3_O_4_ magnetic nanoparticles modified with 3-glycidoxypropyltrimethoxysilane and glycine	158.00	[86]
Calcined titanate nanotubes	133.33	[87]
Gold nanoparticles loaded on activated carbon	104.00–185.00	[88]
Silver nanoparticles loaded on activated carbon	71.43	[89]
Palladium nanoparticles loaded on activated carbon	75.40	[89]
Magnetic halloysite nanotubes/iron oxide composites	18.44	[90]
Zinc molybdate nanoparticles	217.86	[22]
Molybdenum trioxide nanoparticles (hexagonal and orthorhombic phases)	122.50	[64]
Molybdenum trioxide nanorods and stacked nanoplates	152.00	This work
